# Why Does the *GW* Approximation Give Accurate Quasiparticle Energies? The Cancellation of Vertex Corrections Quantified

**DOI:** 10.1021/acs.jpclett.4c03126

**Published:** 2024-12-13

**Authors:** Arno Förster, Fabien Bruneval

**Affiliations:** † Theoretical Chemistry, Vrije Universiteit Amsterdam, De Boelelaan 1105, 1081 HV Amsterdam, The Netherlands; ‡ Université Paris-Saclay, CEA, Service de recherche en Corrosion et Comportement des Matériaux, SRMP, 91191 Gif-sur-Yvette, France

## Abstract

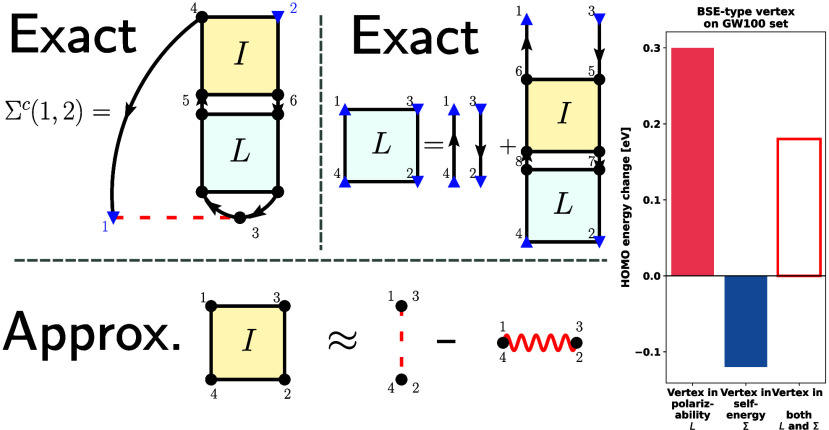

Hedin’s *GW* approximation to the electronic self-energy has been impressively successful in calculating quasiparticle energies, such as ionization potentials, electron affinities, or electronic band structures. The success of this fairly simple approximation has been ascribed to the cancellation of the so-called vertex corrections that go beyond the *GW* approximation. This claim is mostly based on past calculations using vertex corrections within the crude local-density approximation. Here, we explore a wide variety of nonlocal vertex corrections in the polarizability and the self-energy, using first-order approximations or infinite summations to all orders. In particular, we use vertices based on statically screened interactions like in the Bethe–Salpeter equation. We demonstrate on realistic molecular systems that the two vertices in Hedin’s equation essentially compensate. We further show that consistency between the two vertices is crucial for obtaining realistic electronic properties. We finally consider increasingly large clusters and extrapolate that our conclusions about the compensation of the two vertices would hold for extended systems.

The spectroscopic properties of many-electron systems are often described in terms of effective equations for single- and two-particle Green’s functions first formulated by Hedin.^1^ Hedin’s equations start from the Dyson equation for the single-particle Green’s function *G* and express the corresponding self-energy Σ in terms of a dynamically screened electron–electron interaction *W* and a vertex function Γ.

Practical calculations must approximate the vertex function. The most drastic of these approximations is the *GW* approximation (GWA),^1−3^ in which the vertex function is reduced to delta functions. First applied to extended systems^1,4−10^ and later to small metal clusters^11−13^ and molecules,^14−21^ it is by now widely used to describe quasiparticle (QP) levels and band structures in systems as diverse as complex molecules,^22−24^ molecule–metal interfaces,^25−28^ dye-sensitized solar cells,^29−31^ or Moiré materials.^32−34^

In weakly correlated systems, the GWA is relatively accurate for two reasons. First, the dynamical screening of the electron–electron interaction at large distances captures a significant source of electron correlation.^35,36^ While this seems natural in extended systems, it is remarkable that the GWA often gives highly accurate QP energies in atoms and molecules with sometimes only a few electrons.^20,37−39^ This hints at major cancellations between higher-order terms in the self-energy as a second reason for the success of the *GW* approximation. However, despite numerous studies,^16,40−86^ these cancellations are still poorly understood. The partial cancellation of vertex corrections in *W* and in Σ has first been demonstrated for aluminum^40^ and silicon.^42,43,52^ While vertex corrections improve fully self-consistent *GW* (sc*GW*) band gaps and satellites,^45,59,60,63,67,79,87,88^ almost all *GW* calculations replace the interacting *G* by an effective non-interacting *G*^(0)^ that may be judiciously chosen to achieve high accuracy for molecular QP energies.^86,89−91^ Many authors argue that partial cancellations of vertex corrections in *W* and Σ in combination with the QP approximation to *G* are another reason for the success of the GWA in practice,^92^ but this subject is debated.

Including the very same vertex consistently in *W* and Σ allows one to quantify these cancellations rigorously. Following this strategy, previous work has demonstrated the Hartree–Fock (HF) vertex to improve over *GW* QP excitations and satellites in atoms and small molecules.^65,74,81^ The resulting self-energy is free of self-screening^93,94^ but comes with the disadvantage that its beyond-*GW* contribution is expanded in terms of the bare Coulomb interaction instead of the screened one. Especially in larger systems where screening effects are potentially strong, a screened TDHF vertex should be more realistic. Patterson has recently performed such calculations,^85,95^ albeit within the Tamm–Dancoff approximation (TDA) in *L* and Σ. Within the TDA, the same vertex has also been used by Cunningham et al.^68,96^ within quasiparticle self-consistent *GW* (qs*GW*)^92,97−99^ but without any vertex correction in Σ.

We build on these works and further explore the maze of vertex corrections, which is still mostly unmapped. Our quantitative conclusions are based on well-established molecular benchmarks where accurate wave function method-based results offer unambiguous references. We consistently include bare and screened exchange vertices in *W* and Σ. The TDA is known to be a severe approximation in RPA-based *GW* calculations,^100^ and we avoid it here. For a wide range of molecules, including one- and two-dimensional models of graphene and passivated silicon clusters, we demonstrate far-reaching cancellations of vertex corrections, rationalizing the success of the *GW* approximation from small molecules to extended systems.

As shown in Figure 1a, we write the self-energy in the form^101−104^
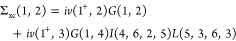
1where integers *n* = (**r**_*n*_, σ_*n*_, *t*_*n*_) collects spatial coordinates, spin and time, *v* is the usual two-point Coulomb interaction, and  is the four-point irreducible kernel. Integration over repeated indices is implied. In the following, we focus on closed shells only and therefore assume spin compensation. As shown in Figure 1b, the two-particle correlation function *L* is obtained through the solution of a Bethe–Salpeter equation (BSE)
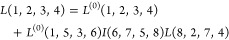
2where we introduced the non-interacting correlation function  and the very same kernel *I* as in Σ appears. Complemented with the Dyson equation for *G*, eqs 1 and 2 yield a self-consistent scheme that is completely equivalent to Hedin’s equations.^105^ This scheme has the major advantage that three-point vertex Γ appears only implicitly, and its explicit calculation is effectively replaced by the solution of the BSE in eq 2.

**Figure 1:**
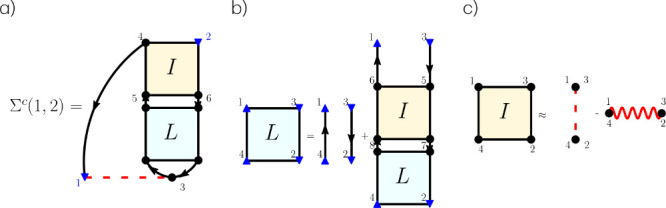
Diagrammatic representation of (a) the correlation part of the exact self-energy, (b) two-particle correlation function *L*, and (c) the approximate kernel we use in this work. Dotted lines denote Coulomb interaction *v*, and the wiggly line is statically screened Coulomb interaction *W*_0_.

We follow previous work^65,70,71,74,81,90^ and exclusively work with a Hartree–Fock (HF) Green’s function
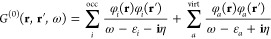
3expressed in terms of HF orbitals φ and HF eigenvalues ε. The indices *i*, *j*, *k*, ..., denote occupied states, and the indices *a*, *b*, *c*, ..., unoccupied (or virtual) states. η is an infinitesimal positive real number. Because HF is diagrammatic, arbitrariness in the choice of *G*^(0)^ is avoided. Moreover, for small molecules, HF orbitals are known to be close to true Dyson orbitals.^106^ For the kernel we choose, the approximation (Figure 1c)
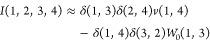
4When *W*_0_ = 0, the GWA is recovered, and when *W*_0_ = *v*, one obtains the time-dependent HF (TDHF) self-energy.^81^ Another possibility is to set *W*_0_ = *W*(ω = 0), where *W* denotes the screened Coulomb interaction calculated within the random-phase approximation (RPA). *L* then turns into the usual BSE implemented in many electronic structure codes, with the important difference being that it is constructed with HF eigenvalues instead of the *GW* ones. When a static approximation to *I* is chosen, only the electron–hole part of *L* contributes to Σ. Equation 2 turns into a function of a single frequency that can be solved exactly by diagonalization in the particle–hole representation.^107^ This part of *L* is^108^
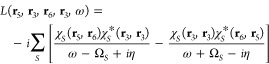
5where the Ω_*S*_ terms are the neutral excitation energies of the system and the amplitudes
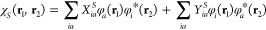
6are expressed in terms of resonant and antiresonant transition matrix elements *X* and *Y*, respectively. We use notations for *X* and *Y* that are consistent with those used in the usual solution of Casida’s equations.^109^ The correlation part of the self-energy can now be written as Σ = Σ^*o*^ + Σ^*v*^ with the contributions
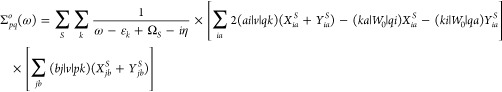
7and
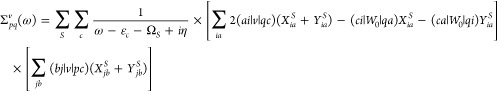
8The factor of 2 comes from spin summation and is absent in the exchange terms. The four-center integrals for *v* and *W*_0_ are defined with the chemists’ notation:
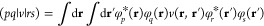
9For a detailed derivation, see the Supporting Information. Other authors have already used this scheme presented there with the TDHF kernel,^65,66,70,81^ and we extend it here by using a screened exchange kernel. On the basis of the prior knowledge of BSE success and the TDHF mixed performance to describe neutral excitations,^110^ we expect this improvement to be significant. The Dyson-like structure of the equations ensures that the kernel is consistently included to infinite order in *L* and hence in Σ. It adds diagrams to the self-energy that describe electron–hole interactions and are important at short interelectronic distances.^111^ We go beyond approaches that include the vertex in Σ to first order only,^42,47,49^ leading for instance to *G*3*W*2 vertex corrections^59,62,63,86^ and approximations like its completely statically screened version,^55,78^ SOSEX,^38,57,112^ or subsets of *G*3*W*2.^56,60,72^ Using different kernels in *L* and Σ is possible, but we show here that the kernels should be kept consistent. The different approximations used in this work are summarized in Table 1.

**Table 1:** Summary of the Different Infinite-Order Approximations Used in This Work

*I*(6, 7, 5, 8) in *L*	*L*	*I*(3, 5, 4, 6) in Σ	Σ	vernacular name
0	*L*^(0)^	*v*(3, 6)δ(3, 4)δ(5, 6) – *v*(3, 4)δ(3, 6)δ(4, 5)	Σ^PT2^	PT2, GF2, or 2-Born
*v*(6, 8)δ(6, 5)δ(7, 8)	*L*^TDH^	*v*(3, 6)δ(3, 4)δ(5, 6)	*GW@L*^TDH^	standard *GW*
*v*(6, 8)δ(6, 5)δ(7, 8) – *v*(6, 5)δ(6, 8)δ(7, 5)	*L*^TDHF^	*v*(3, 6)δ(3, 4)δ(5, 6)	*GW@L*^TDHF^	*GW* with TDHF screening
*v*(6, 8)δ(6, 5)δ(7, 8) – *W*_0_(6, 5)δ(6, 8)δ(7, 5)	*L*^BSE^	*v*(3, 6)δ(3, 4)δ(5, 6)	*GW@L*^BSE^	*GW* with BSE screening
*v*(6, 8)δ(6, 5)δ(7, 8) – *v*(6, 5)δ(6, 8)δ(7, 5)	*L*^TDHF^	*v*(3, 6)δ(3, 4)δ(5, 6) – *v*(3, 4)δ(3, 6)δ(4, 5)	Σ^TDHF^	TDHF self-energy
*v*(6, 8)δ(6, 5)δ(7, 8) – *W*_0_(6, 5)δ(6, 8)δ(7, 5)	*L*^BSE^	*v*(3, 6)δ(3, 4)δ(5, 6) – *W*_0_(3, 4)δ(3, 6)δ(4, 5)	Σ^BSE^	BSE self-energy

In the following, we discuss the numerical results. We first test different vertex-corrected schemes on the GW100 test set of first molecular ionization potentials.^113^ We perform all calculations with MOLGW^114^ and BAND^115,116^ using the def2-qzvpp basis set and use the corresponding CCSD(T) values from ref (117) as reference.

Figure 2 shows their error distributions of several vertex-corrected schemes compared with CCSD(T) together with mean absolute deviations (MADs) and root-mean-square deviations (RMSDs). The leftmost violin in Figure 2a shows the errors of *GW*@RPA. The next two violins show the errors for the *GW* self-energy with *L* calculated with TDHF and BSE, respectively. The final two plots show the results for Σ^TDHF^*@L*^TDHF^ and Σ^BSE^*@L*^BSE^, respectively, which both include the vertex in eq 4 consistently to infinite order in *L* and Σ. All four vertex-corrected schemes give major improvements over *GW*. This is also true for *GW@L*^TDHF^ and *GW@L*^BSE^ that include only the vertex in *L*. Therefore, our results temper the strong conclusions of Lewis et al.,^71^ who claimed that the efforts to improve the screening part of the self-energy are making the results worse. However, *GW@L*^TDHF^ and *GW@L*^BSE^ (to a lesser extent) lead to major errors in some molecules. The kernel in Σ balances the sizable effect of the kernel in *L*, leading to consistent improvements over *GW*@RPA.

**Figure 2:**
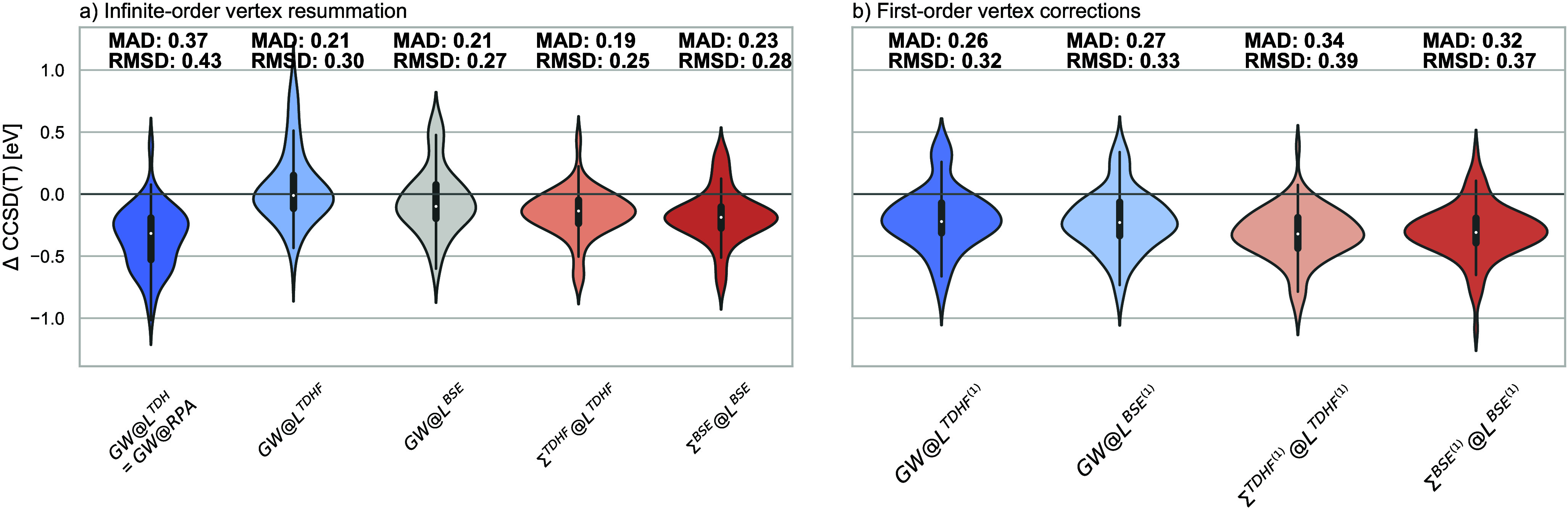
Errors of *GW* and several vertex-corrected schemes with respect to CCSD(T) in electronvolts of the HOMO of the molecules contained in the GW100 set for (a) infinite vertex resummation and (b) first order only.

To understand this behavior, we show that the vertex corrections systematically have opposite signs in Figure 3 and therefore partially and sometimes completely cancel. The orange bars show the magnitude of the vertex correction beyond TDH in *L* (corresponding to the third violin in Figure 2a), and the blue bars show the magnitude of the vertex correction in Σ beyond *GW* (corresponding to the last violin in Figure 2a). The red boxes show the difference between *GW*@RPA and Σ^BSE^*@L*^BSE^, which is the sum of the blue and orange bars. The BSE kernel describes the electron–hole interaction missing in *GW*@RPA that stabilizes the cation and therefore decreases the HOMO energy. In some cases exceeding 0.6 eV, this effect is sizable for most molecules in GW100, and frequently, the HOMO energy is overcorrected. The vertex correction in Σ has the opposite effect and reduces the HOMO energy further. The effect of the vertex is generally stronger for *L* than for Σ, and therefore, the combination of both vertex corrections lowers the HOMO. Both vertices combined lead to the observed improvement in Σ^BSE^*@L*^BSE^ over *GW*@RPA.

**Figure 3:**
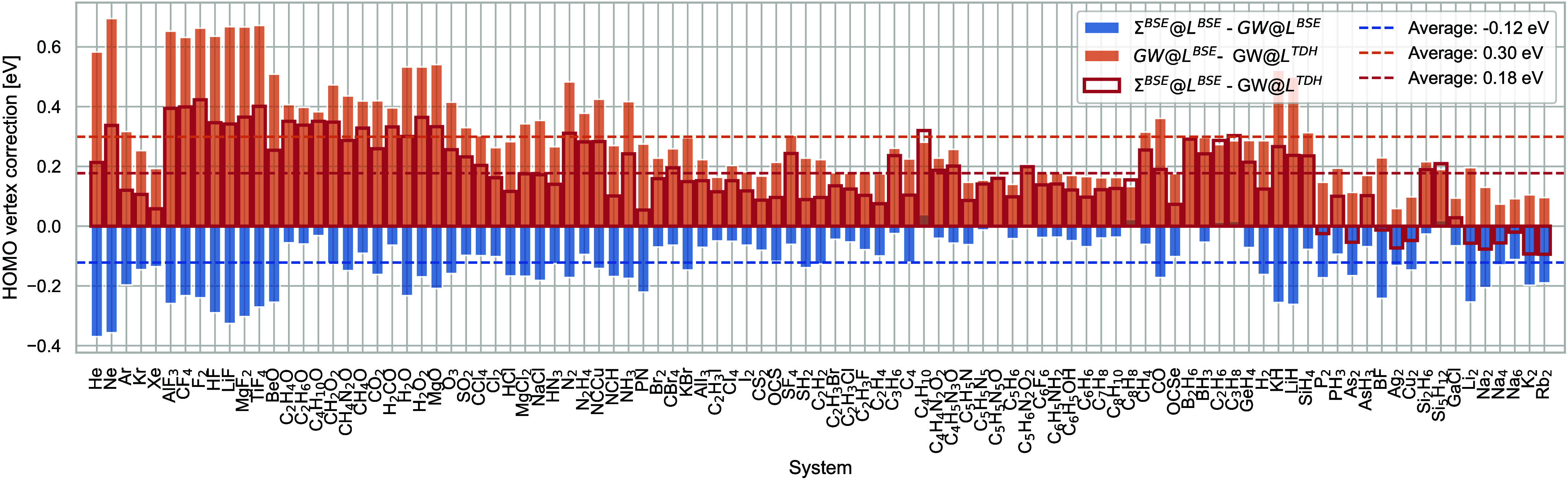
Vertex corrections in electronvolts of the highest occupied molecular orbital of the molecules in the GW100 set. In addition to the rare gases, the molecules are sorted by decreasing electronegativity of the element most represented in the HOMO.

As shown in Figure S2, a similar picture is obtained for the TDHF screening and self-energy approximations. Our results qualitatively agree with those of ref (81). With average values of 0.41 and −0.21 eV, the effect of the individual vertex corrections in *L* and Σ, respectively, is significantly larger. However, at 0.2 eV on average, the combined effect of the vertex correction is comparable to the BSE. The BSE vertex correction accounts for higher-order vertex diagrams not included in Σ^TDHF^*@L*^TDHF^. The smaller magnitudes of the vertex corrections in *L* and Σ with the BSE vertex indicate further cancellations between these higher-order diagrams.

Further insight into the cancellation of vertex corrections is provided in Figure 2b, where we show the errors of the same vertex-corrected schemes as in Figure 2a, but in all cases truncated to first order. For polarizability, this means that RPA screening is modified by including only one diagram of first order in *W*_0_.^46,47,62^ Upon inclusion of the same vertex diagram in Σ, the SOSEX self-energy is obtained with the bare vertex,^86^ and the screened vertex leads to a second-order term similar to SOSEX but with the bare *v* replaced by the statically screened one. In this scheme, the vertex correction is consistent in *L* and Σ because the next-to-leading order diagram is added to both quantities. See section S3 of the Supporting Information for detailed derivations.

Including the kernel to first order only has generally a much smaller effect than the infinite-order resummations (0.17 eV on average vs 0.41 eV for *L* and −0.13 vs −0.21 eV for Σ) As shown in Figures S3 and S4, adding the same vertex correction to *W* and Σ results in HOMO energies almost indistinguishable from *GW*@RPA. The same conclusion has already been drawn on the basis of the results for the band gap of silicon^42^ and for a one-dimensional semiconductor,^44^ and we confirm here its validity for molecules. This almost complete cancellation of the next-to-leading order terms in *L* and Σ rationalizes the good performance of *GW*@RPA for calculating QP energies.

Finally, in Figure 4, we show the magnitude of the different BSE vertex corrections for the HOMO and LUMO energies of molecules of systematically increasing size: linear acenes ranging from a single benzene ring (C_6_H_6_) to hexacene (C_26_H_16_), coronene, and circumcoronene, as well as passivated silicon clusters with ≤37 silicon atoms. All of these calculations have been performed with different Dunning-type basis sets ranging from double-ζ to quadruple-ζ quality.^118^ For details, see the Supporting Information. For the BSE vertex, we find the magnitude of the vertex correction to be almost independent of system size and with ∼0.1 eV to be rather small. The effect on the LUMO is with 0.2 eV approximately twice as large. This observation is consistent with ref (70). While initially increasing, the magnitude of the vertex correction stays approximately constant for the linear acenes and silicon clusters. We also notice that the first-order truncation of Σ^BSE^ is always a good approximation.

**Figure 4:**
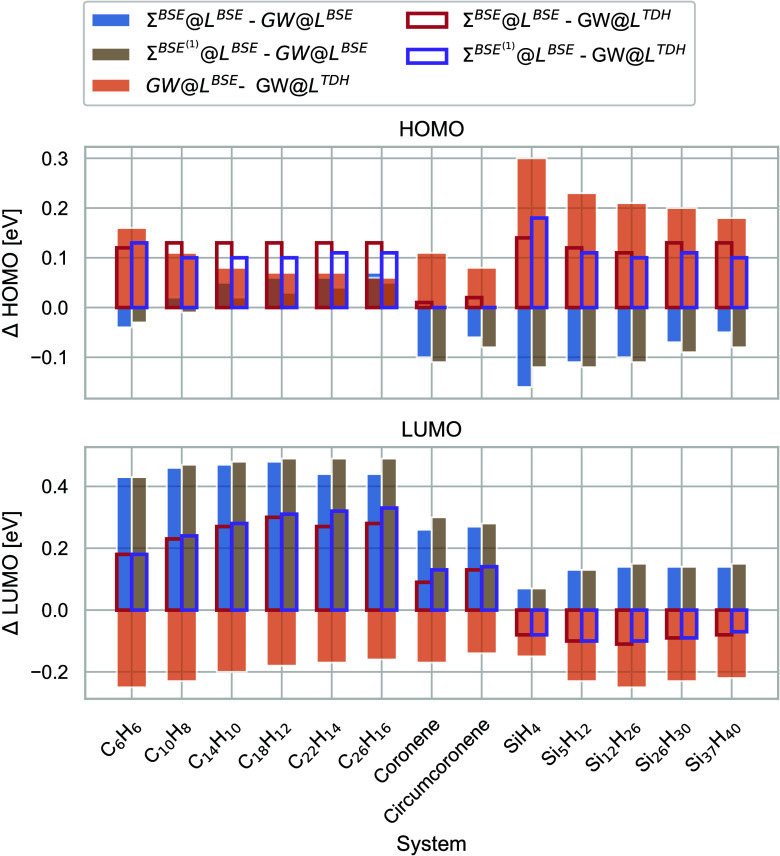
Screened exchange vertex corrections in electronvolts of the HOMO (top) and LUMO (bottom) for linear acenes, nonlinear acenes, and passivated silicon clusters of increasing size.

Figure 5 shows the same information for the TDHF vertex. As for GW100, the magnitudes of the individual vertex corrections in Σ and *L* are larger than those for the BSE vertex. Also, the total vertex correction is much larger than for the BSE vertex. Moreover, we observe, especially for the linear acenes, that the infinite-order resummation of the TDHF vertex in Σ^TDHF^ leads to a rapidly increasing vertex correction for the HOMO. At the same time, its first-order approximation (SOSEX) goes to almost zero. The opposite can be observed for the LUMO. This inconsistency indicates the importance of screened vertices for larger systems.

**Figure 5:**
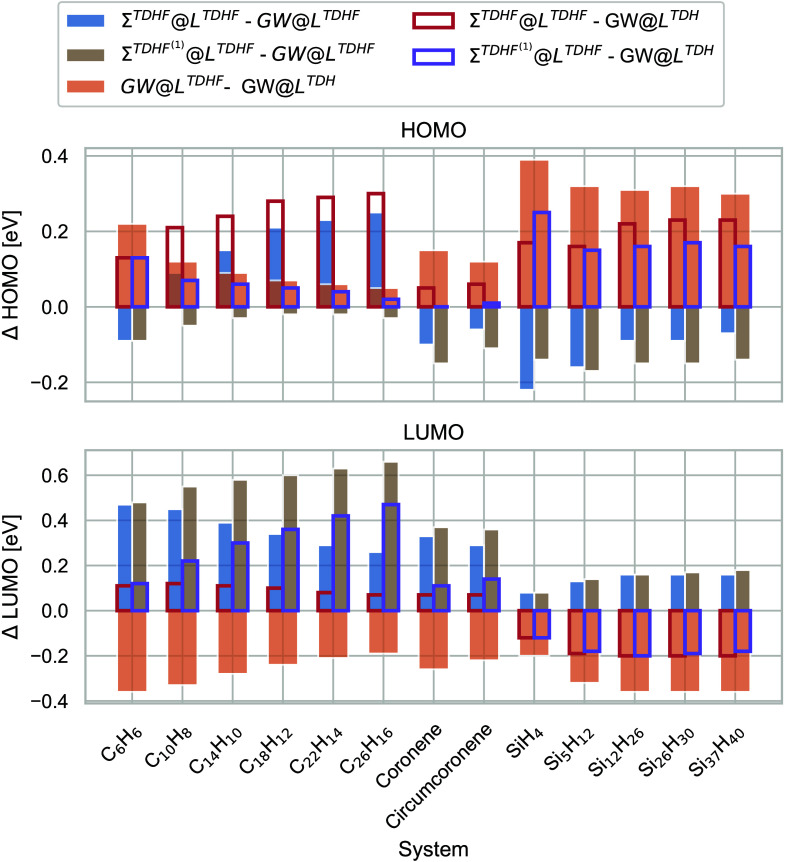
Bare exchange vertex corrections in electronvolts of the HOMO (top) and LUMO (bottom) for linear acenes, nonlinear acenes, and passivated silicon clusters of increasing size.

In conclusion, several vertex-corrected schemes have been investigated over the past several decades to improve over the simple GWA for QP energies. Cancellations between vertex corrections were observed early on for simple (model) systems.^40,43,44^ Despite immense implications for practical *GW* calculations, these results have never been generally confirmed using realistic, nonlocal vertices.

With this work, we have filled this gap. To rationalize the success of the GWA for calculating QP energies, we have investigated several vertex corrections beyond the GWA. We benchmarked these methods for systems ranging from small and medium molecules in the GW100 set, over linear and nonlinear acenes, to silicon clusters. We have used the TDHF vertex as obtained from the HF self-energy, which adds infinite-order particle–hole diagrams to *L* and Σ as well as a BSE vertex, which statically screens these diagrams. Especially for larger molecules, using a screened vertex correction becomes decisive.

By restricting infinite-order vertex summation to first order only, we have also performed calculations that include only the next-to-leading order correction to *L* and Σ. Both corrections effectively cancel for HOMO QP energies, suggesting an order-by-order expansion of *L* and Σ beyond *GW*@RPA to be inefficient. Despite being of low order in perturbation theory, the GWA accounts for the most important signatures of electron correlation for charged excitation.

We have rationalized why schemes that add a vertex correction to either the response function or the self-energy have been unsuccessful.^71,86^ The cancellations between these vertices are far-reaching, and they both must be included to obtain systematic improvements over *GW*. To improve over *GW*@RPA, infinite-order resummations of the vertex function are needed in both *L* and Σ. In the future, dynamic vertex corrections could be explored. These would allow for the inclusion of the yet missing particle–particle channel to the self-energy that is important in the strongly correlated regime.^77,119^

## Supporting Information Available

Additional data (TXT)
Detailed derivations and discussions of all equations, additional computational details, and all QP energies calculated in this work (PDF)
Transparent Peer Review report available (PDF)
