# Supplementary material for: Why Does the GW Approximation Give Accurate Quasiparticle Energies? The Cancellation of Vertex Corrections Quantified
Source: J Phys Chem Lett. 2024 Dec 13;15(51):12526–34. doi: 10.1021/acs.jpclett.4c03126 (PMC11684030; doi:10.1021/acs.jpclett.4c03126)
Supplement: Supplementary file 2 — jz4c03126_si_002.pdf [file jz4c03126_si_002.pdf]

Supporting information to "Why does the GW approximation give accurate quasiparticle energies? The cancellation of vertex corrections quantified"

Arno Förster[1] and Fabien Bruneval[2]

[1)] *Theoretical Chemistry, Vrije Universiteit Amsterdam, De Boelelaan 1105, 1081 HV Amsterdam, The Netherlands*

[2)] *Université Paris-Saclay, CEA, Service de recherche en Corrosion et Comportement des Matériaux, SRMP, 91191 Gif-sur-Yvette, France*

(*Electronic mail: fabien.bruneval@cea.fr)

(*Electronic mail: a.t.l.foerster@vu.nl)

(Dated: 4 December 2024)

CONTENTS

S1. Hedin's equations and notation 2

S2. Alternative expression for the self-energy 3

S2.1. Working equations for Σ 4

S3. First-order vertex in *P* and Σ 6

S3.1. Working equations 7

1. Self-energy 7
2. Polarizability 7

S4. Technical details 14

S5. Further results for GW100 15

S5.1. Basis set convergence for different methods 15

S5.2. Vertex corrections for the HOMO of the molecules in GW100 15

S6. Results for large systems 16

References 20

S1. HEDIN'S EQUATIONS AND NOTATION

Hedin's equations[1] relate the three-point vertex function,

$$\begin{aligned}\tilde{\Gamma}(1,2,3) =& \delta(1,2)\delta(1,3) \\ &+ \tilde{I}(1,5,2,4)L^{(0)}(4,7,5,6)\tilde{\Gamma}(6,7,3) \,,\end{aligned} \tag{1a}$$

the electronic self-energy

$$\Sigma_{xc}(1,2) = iG(1,4)W(1,3^+)\tilde{\Gamma}(4,2,3) \,, \tag{1b}$$

the screened Coulomb interaction

$$W(1,2) = v_c(1,2) + v_c(1,3)P^{(0)}(3,4)W(4,2) \,, \tag{1c}$$

the irreducible polarizability

$$P^{(0)}(1,2) = L^{(0)}(1,4,1,3)\tilde{\Gamma}(3,4,2) \,, \tag{1d}$$

and the Dyson equation for the single-particle Greens-function G

$$G(1,2) = G^{(0)}(1,2) + G^{(0)}(1,3)\Sigma_{xc}(3,4)G(4,2)\ . \tag{1e}$$

Our definition of $L^{(0)}$ follows Romaniello et al.[2]

$$L^{(0)}(1,2,3,4) = -iG(1,4)G(2,3) \tag{2}$$

and $\tilde{I}$ is defined accordingly.[2]

$$\tilde{I}(1,2,3,4) = i\frac{\delta\Sigma_{xc}(1,3)}{\delta G(4,2)}\ . \tag{3}$$

For later reference it is also useful to define

$$I(1,2,3,4) = i\frac{\delta\Sigma(1,3)}{\delta G(4,2)} = \delta(3,4)\delta(5,6)v(3,6) - \tilde{I}(1,2,3,4)\ . \tag{4}$$

For first term in the last equation on the r.h.s. is obtained as the functional derivative of the Hartree self-energy with respect to G. The vertices we consider in this work are obtained as functional derivatives of the Hartree–Fock self-energy,

$$I^{HF}(3,5,4,6) = \delta(3,4)\delta(5,6)v(3,5) - \delta(3,6)\delta(4,5)v(3,4)\ , \tag{5}$$

and of the GW self-energy, respectively, after the usual static approximation and neglecting terms of second order in W,

$$I^{GW}(3,5,4,6) \approx \delta(3,4)\delta(5,6)v(3,6) - \delta(3,6)\delta(4,5)W(\mathbf{r}_3,\mathbf{r}_4,\omega=0)\ . \tag{6}$$

All of the following derivations are valid for all of these vertices and we write it as

$$I(3,5,4,6) \approx \delta(3,4)\delta(5,6)v(3,6) - \delta(3,6)\delta(4,5)W_0(3,4)\ . \tag{7}$$

S2. ALTERNATIVE EXPRESSION FOR THE SELF-ENERGY

According to Strinati[3] Eq. (3.16) (integrating V out)

$$\Sigma_{xc}(1,2) = -i\int d34 v(1^+,3)G(1,4)\frac{\delta G^{-1}(4,2)}{\delta U(3)}\ . \tag{8}$$

U is the total classical potential. The last factor in this expression is the reducible vertex

$$\Gamma(4,2,3) = -\frac{\delta G^{-1}(4,2)}{\delta U(3)} \tag{9}$$

Introducing the Dyson equation for G^{-1},

$$G^{-1}(4,2) = G_0^{-1}(4,2) - U(4)\delta(4,2) - v_H(4)\delta(4,2) - \Sigma_{xc}(4,2) \tag{10}$$

we get

$$\begin{aligned}\Sigma_{xc}(1,2) = i\int d34 v(1^+,3)G(1,4)\delta(4,2)\delta(4,3) \\ + i\int d3456 v(1^+,3)G(1,4)\frac{\delta\left[v_H(4)\delta(4,2)+\Sigma_{xc}(4,2)\right]}{\delta G(5,6)}\frac{\delta G(5,6)}{\delta U(3)}\end{aligned} \tag{11}$$

where one recognizes

$$I(4,6,2,5) = i\frac{\delta\left[v_H(4)\delta(4,2)+\Sigma_{xc}(4,2)\right]}{\delta G(5,6)} \tag{12}$$

and

$$L(5,3,6,3) = -i\frac{\delta G(5,6)}{\delta U(3)} \tag{13}$$

to finally obtain

$$\Sigma_{xc}(1,2) = iv(1^+,2)G(1,2) + i\int d3456 v(1^+,3)G(1,4)I(4,6,2,5)L(5,3,6,3)\ . \tag{14}$$

The same or equivalent expressions have already been presented previously in the literature.[4–7]

S2.1. Working equations for Σ

Using eq. (7) the first term in (14) reduces to

$$\begin{aligned} &i\int d3456 v(1^+,3)G(1,4)\delta(4,2)\delta(6,5)v(4,5)L(5,3,6,3) \\ =&i\int d34 v(1^+,3)G(1,2)v(2,4)L(4,3,4,3)\ . \end{aligned} \tag{15}$$

With $P(4,3) = P(3,4) = L(3,4,3,4) = L(4,3,4,3)$ and $v(2,4) = v(4,2)$ and the definition

$$W(1,2) = v(1,2) + \int d34 v(1,3)P(3,4)v(4,2)$$

this term can be combined with the exchange term in (14) to give the GW approximation,

$$\Sigma_{xc}^{GW}(1,2) = iW(1^+,2)G(1,2)\ .$$

The second term in (15) gives

$$-i\int d3456 v(1^+,3)G(1,4)\delta(4,5)\delta(2,6)v(4,2)L(5,3,6,3) \tag{16}$$

The explicit expression for L is[8]

$$L(\mathbf{r}_5,\mathbf{r}_3,\mathbf{r}_6,\mathbf{r}_7,\omega) = -i\sum_S \left[\frac{\chi_S(\mathbf{r}_5,\mathbf{r}_6)\chi_S^*(\mathbf{r}_7,\mathbf{r}_3)}{\omega-\Omega_S+i\eta} - \frac{\chi_S(\mathbf{r}_3,\mathbf{r}_7)\chi_S^*(\mathbf{r}_6,\mathbf{r}_5)}{\omega+\Omega_S-i\eta} \right] \tag{17}$$

with

$$\chi_S(\mathbf{r}_1,\mathbf{r}_2) = \sum_{ia} X_{ia}^S \varphi_a(\mathbf{r}_1)\varphi_i^*(\mathbf{r}_2) + \sum_{ia} Y_{ia}^S \varphi_i(\mathbf{r}_1)\varphi_a^*(\mathbf{r}_2)\ . \tag{18}$$

To arrive at the working equation given in the main text, we

1. Start from the real space expression (16).
2. orbital-transform the external space indices $\mathbf{r}_1$ and $\mathbf{r}_2$.
3. Introduce the expression of L in real space from Eq. (17)
4. and finally identify the Coulomb integrals that will show up (in chemist's notation).

One then gets the resonant part (corresponding to $+\Omega_S$), which corresponds to occupied k index,

$$\Sigma_{pq}^{o}(\omega) = -\sum_S \sum_k \frac{1}{\omega-\varepsilon_k+\Omega_S-i\eta} \times \left[\sum_{ia} (ka|W_0|qi)X_{ia}^S + (ki|W_0|qa)Y_{ia}^S \right] \times \left[\sum_{jb} (bj|v|pk)(X_{jb}^S+Y_{jb}^S) \right] \tag{19}$$

and a similar expression for the anti-resonant part ($-\Omega_S$),

$$\Sigma_{pq}^{v}(\omega) = -\sum_S \sum_c \frac{1}{\omega-\varepsilon_c-\Omega_S+i\eta} \times \left[\sum_{ia} (ci|W_0|qa)X_{ia}^S + (ca|W_0|qi)Y_{ia}^S \right] \times \left[\sum_{jb} (bj|v|pc)(X_{jb}^S+Y_{jb}^S) \right] \tag{20}$$

with the four-center integrals

$$(pq|v|rs) = \int d\mathbf{r} \int d\mathbf{r}' \varphi_p^*(\mathbf{r})\varphi_q(\mathbf{r})v(\mathbf{r},\mathbf{r}')\varphi_r^*(\mathbf{r}')\varphi_s(\mathbf{r}')\ . \tag{21}$$

This can be combined with the GW portion of the self-energy to obtain the expression in the main text.

As a consistency check, we notice that one should be able to recover the SOX part of the PT2 self-energy:

$$\Sigma^{o}_{pq}(\omega) = -\sum_{ija} \frac{(pj|ai)(qi|aj)}{\omega - \varepsilon_i - \varepsilon_j + \varepsilon_a - i\eta} \tag{22}$$

$$\Sigma^{v}_{pq}(\omega) = -\sum_{iab} \frac{(pb|ai)(qa|bi)}{\omega - \varepsilon_i + \varepsilon_a + \varepsilon_b + i\eta} \tag{23}$$

when L is replaced by L_0. The limit $L \to L_0$ is obtained when the S excitation corresponds to $j \to b$ transition only. One therefore has to make the substitutions

$$X^S_{ia} = \delta_{ij}\delta_{ab} \tag{24}$$

$$Y^S_{ia} = 0 \tag{25}$$

$$\Omega_S = \varepsilon_b - \varepsilon_j \,. \tag{26}$$

Similarly, for the anti-resonant part, an S excitation corresponds to $b \to j$ transition only. One therefore has to make the substitutions

$$X^S_{ia} = \delta_{ij}\delta_{ab} \tag{27}$$

$$Y^S_{ia} = 0 \tag{28}$$

$$\Omega_S = \varepsilon_j - \varepsilon_b \,. \tag{29}$$

S3. FIRST-ORDER VERTEX IN P AND Σ

Hedin's equations are considerably simplified when (1a) is linearized as

$$\tilde{\Gamma}(1,2,3) \approx \delta(1,2)\delta(1,3) + \tilde{I}(1,5,2,4)L^{(0)}(4,3,5,3) \,. \tag{30}$$

Inserting (30) into (1d) and into (1b) allows us to eliminate the three-point vertex from both equations and one obtains

$$\begin{aligned}\Sigma_{xc}(1,2) =& iG(1,2)W(1,2) \\ &+ iG(1,4)W(1,3)\tilde{I}(4,5,2,6)L^{(0)}(6,3,5,3)\end{aligned} \tag{31}$$

and

$$\begin{aligned}P^{(0)}(1,2) =& L^{(0)}(1,2,1,2) \\ &+ L^{(0)}(1,4,1,3)\tilde{I}(3,5,4,6)L^{(0)}(6,2,5,2) \,.\end{aligned} \tag{32}$$

The latter equation is recognized as a Bethe-Salpeter equation (BSE) for the irreducible polarizability up to first order in $\tilde{I}$. Together with (1e) and (1c), (31) and (35) form a system of four coupled equations which we refer to as linearized Hedin's equations.

S3.1. Working equations

We now present the explicit working equations for Σ and P using the vertex (7) on the imaginary frequency axis.

1. Self-energy

The expression for the self-energy is derived by inserting the vertex $W(3,4)\delta(3,6)\delta(4,5)$ into (31). As shown in ref. 9 this leads to the fully dynamical $G3W2$ self-energy and replacing the dynamical vertex with the static $\tilde{I}^{TDHF}$ leads to the well-known SOSEX contribution to the self-energy[9,10]

$$\Sigma = -G(1,4)W(1,3)G(4,3)G(3,2)W_0(4,2) \ . \tag{33}$$

In orbital basis and on the imaginary axis, it can be expressed as[9]

$$\Sigma^{\text{SOSEX}}_{pq}(\mu+i\omega) = -\frac{1}{2\pi}\int d\omega'' \sum_u\sum_v\sum_w (f_u - f_v)\frac{(wv|W_0|pu)(qw|W_p(i\omega'')|uv)}{(i\omega''+\varepsilon_u-\varepsilon_v)(\mu+i\omega+i\omega''-\varepsilon_w)} \ , \tag{34}$$

where we have chosen to use the occupation numbers f_u which are either 0 or 1 do distinguish between occupied and unoccupied states. μ is the chemical potential which is placed in the middle of the highest occupied and lowest unoccupied KS state.

2. Polarizability

Combining (35) with (7), we obtain

$$\begin{aligned} P^{(0)}(1,2) =& L^{(0)}(1,2,1,2) - L^{(0)}(1,4,1,3)\delta(3,6)\delta(4,5)W_0(3,4)L^{(0)}(6,2,5,2) \\ =& L^{(0)}(1,2,1,2) - L^{(0)}(1,4,1,3)W_0(3,4)L^{(0)}(3,2,4,2) \\ =& P^{(0)^{RPA}}(1,2) + P^{(0)^{(1)}}(1,2) \end{aligned} \tag{35}$$

We notice, that we can here restrict ourselves to the polarizability $P^{(0)}$ as opposed to the full L.

a. RPA polarizability on the real frequency axis. We start with the first term on the r.h.s. to (35) which is the RPA irreducible polarizability. We use the following definitions of Fourier transforms

$$G(\omega) = \int d\tau e^{i\omega\tau}G(\tau) \tag{36}$$

$$G(\tau) = \frac{1}{2\pi}\int d\omega e^{-i\omega\tau}G(\omega) \ , \tag{37}$$

Substituting $\tau = t_1 - t_2$ and omitting spatial arguments for brevity, we obtain for the RPA polarizability

$$\begin{aligned}
P^{(0)^{RPA}}(\omega) &= \int d\tau e^{i\omega\tau} P^{(0)^{RPA}}(\tau) \\
&= -i \int d\tau e^{i\omega\tau} G(\tau) G(-i\tau) \\
&= -\frac{i}{(2\pi)^2} \int d\tau e^{i\omega\tau} \int d\omega' d\omega'' e^{i\tau(\omega+\omega''-\omega')} G(\omega') G(\omega'') \\
&= -\frac{i}{2\pi} \int d\omega' \delta(\omega + \omega'' - \omega') G(\omega') G(\omega'') \\
&= -\frac{i}{2\pi} \int d\omega' G(\omega') G(\omega' - \omega) \; .
\end{aligned} \tag{38}$$

With the definition of $G^{(0)}$ from the main text,

$$G^{(0)}(\mathbf{r}, \mathbf{r}', \omega) = \sum_{i}^{\text{occ}} \frac{\varphi_i(\mathbf{r})\varphi_i(\mathbf{r}')}{\omega - \varepsilon_i - i\eta^+} + \sum_{a}^{\text{virt}} \frac{\varphi_a(\mathbf{r})\varphi_a(\mathbf{r}')}{\omega - \varepsilon_a + i\eta^+} \; . \tag{39}$$

we get

$$\begin{aligned}
P^{(0)^{RPA}}(\omega) &= -\frac{i}{2\pi} \int d\omega' G(\omega') G(\omega' - \omega) \\
&= -\frac{i}{2\pi} \lim_{\eta \to 0^+} \int d\omega' \left\{ \sum_{ij} \frac{1}{\omega' - i\eta - \varepsilon_i} \frac{1}{\omega' - \omega - i\eta - \varepsilon_j} \right. \\
&+ \sum_{ia} \frac{1}{\omega' - i\eta - \varepsilon_i} \frac{1}{\omega' - \omega + i\eta - \varepsilon_a} \\
&+ \sum_{ia} \frac{1}{\omega' + i\eta - \varepsilon_a} \frac{1}{\omega' - \omega - i\eta - \varepsilon_i} \\
&\left. + \sum_{ab} \frac{1}{\omega' + i\eta - \varepsilon_a} \frac{1}{\omega' - \omega + i\eta - \varepsilon_b} \right\} \; .
\end{aligned} \tag{40}$$

The first and last term won't contribute any poles. For the second term we chose a clockwise contour in the lower half of the complex plane and for the third term we chose a anticlockwise contour in the upper half of the complex plane. We then obtain in the particle-hole basis

$$\begin{aligned}
P^{(0)^{RPA}}_{ia,jb}(\omega) &= -\frac{i}{2\pi} \lim_{\eta \to 0^+} \delta_{ab}\delta_{ij} \left\{ \frac{2\pi i}{-\omega + 2i\eta + \varepsilon_i - \varepsilon_a} - \frac{2\pi i}{-\omega - 2i\eta - \varepsilon_i + \varepsilon_a} \right\} \\
&= \lim_{\eta \to 0^+} \delta_{ab}\delta_{ij} \left\{ \frac{1}{\omega - (\varepsilon_a - \varepsilon_i - 2i\eta)} - \frac{1}{\omega + (\varepsilon_a - \varepsilon_i - 2i\eta)} \right\} \; .
\end{aligned} \tag{41}$$

b. RPA polarizability on the Imaginary frequency axis On the imaginary frequency axis, we can write

$$
\begin{aligned}
P^{(0)^{RPA}}(i\omega) =& i\int e^{-i\omega\tau}P(\tau)d\tau \\
=& -\left(\frac{1}{2\pi}\right)^2\int e^{-i\tau(-\omega+\omega'-\omega'')}G(i\omega'+\mu)G(i\omega''+\mu)d\omega'\omega''d\tau \\
=& -\frac{1}{2\pi}\int d\omega' G(i\omega'+\mu)G(i\omega'-i\omega+\mu)
\end{aligned}
$$

Using the explicit form of G, we get

$$
\begin{aligned}
P^{(0)^{RPA}}_{pqrs}(i\omega) =& -\frac{1}{2\pi}\int d\omega'\delta_{ps}\delta_{qr}\frac{1}{i\omega'+\mu-\varepsilon_p}\frac{1}{i\omega'-i\omega+\mu-\varepsilon_q} \\
=& -\delta_{ps}\delta_{qr}(f_p-f_q)\frac{1}{\varepsilon_p-\varepsilon_q+i\omega}
\end{aligned}
$$

Therefore,

$$
\begin{aligned}
P^{(0)^{RPA}}_{ia,jb}(i\omega) =& \delta_{ij}\delta_{ab}\left\{\frac{1}{\varepsilon_a-\varepsilon_i+i\omega}-\frac{1}{\varepsilon_i-\varepsilon_a+i\omega}\right\} \\
=& \delta_{ij}\delta_{ab}\left\{\frac{1}{\varepsilon_a-\varepsilon_i+i\omega}+\frac{1}{\varepsilon_a-\varepsilon_i-i\omega}\right\} \\
=& \delta_{ia}\delta_{jb}\frac{1}{\varepsilon_a-\varepsilon_i+i\omega}+c.c.
\end{aligned}
\tag{42}
$$

This shows that the real frequency formulas can be converted to imaginary frequency ones by performing the substitution $\omega-2i\eta\rightarrow -i\omega$.

c. First-order vertex in P. For the beyond-RPA term, we choose the definitions

$$
\begin{aligned}
\tau =& t_1-t_2 \\
\tau' =& t_3-t_4 \\
\tau'' =& t_1-t_3\ .
\end{aligned}
$$

we obtain the following structure in real time,

$$
P^{(0)^{(1)}}(\tau) = -i^2\int d\tau' d\tau'' G(\tau'')W(\tau')G(\tau-\tau'')G(-\tau+\tau''+\tau')G(-\tau'-\tau'') \tag{43}
$$

Fourier transforming P and Fourier transforming all the G and W gives

$$
\begin{aligned}
P^{(0)^{(1)}}(\omega) =& \int d\tau e^{i\omega\tau} P^{(0)^{(1)}}(\tau) \\
=& -\frac{i^2}{(2\pi)^5} \int d\tau d\tau' d\tau'' \int d\omega' d\omega'' d\omega_1 d\omega_2 d\omega_3 e^{i\omega\tau} e^{-i\omega''\tau''} e^{-i\omega'\tau'} \\
& e^{-i\omega_1(\tau-\tau'')} e^{-i\omega_2(-\tau+\tau''+\tau')} e^{-i\omega_3(-\tau'-\tau'')} G(\omega'')W(\omega')G(\omega_1)G(\omega_2)G(\omega_3) \\
=& -\frac{i^2}{(2\pi)^5} \int d\omega' d\omega'' d\omega_1 d\omega_2 d\omega_3 \int d\tau d\tau' d\tau'' e^{i(\omega-\omega_1+\omega_2)\tau} e^{i(-\omega'-\omega_2+\omega_3)\tau'} e^{i(-\omega''+\omega_1-\omega_2+\omega_3)\tau''} \\
& G(\omega'')W(\omega')G(\omega_1)G(\omega_2)G(\omega_3)
\end{aligned}
\tag{44}
$$

We get

$$
\begin{pmatrix} \omega \\ \omega' \\ \omega'' \end{pmatrix} = \begin{pmatrix} -1 & 1 & 0 \\ 0 & -1 & 1 \\ 1 & -1 & 1 \end{pmatrix} \begin{pmatrix} \omega_1 \\ \omega_2 \\ \omega_3 \end{pmatrix} \tag{45}
$$

and therefore

$$
\begin{pmatrix} \omega_1 \\ \omega_2 \\ \omega_3 \end{pmatrix} = \begin{pmatrix} 0 & -1 & 1 \\ 1 & -1 & 1 \\ 1 & 0 & 1 \end{pmatrix} \begin{pmatrix} \omega \\ \omega' \\ \omega'' \end{pmatrix} \tag{46}
$$

We therefore obtain

$$
P^{(0)^{(1)}}(\omega) = \frac{1}{(2\pi)^2} \int d\omega' d\omega'' G(\omega'')W(\omega')G(\omega''-\omega')G(\omega-\omega'+\omega'')G(\omega+\omega'') \,. \tag{47}
$$

This expression is equivalent to the one given in the supporting information of reference 11. Even with a dynamical vertex $W(\omega')$ it is possible to perform the integration over ω'' analytically by inserting the explicit expression for $G^{(0)}$ (39) into the integral

$$
\int d\omega'' G(\omega'')G(\omega''-\omega')G(\omega-\omega'+\omega'')G(\omega+\omega'') \,.
$$

One finds that the contributions from all Green's functions with all four indices being occupied or virtual are zero. For the other terms we find:

- Contributions with a single virtual and three occupied G:

  $$
  \int d\omega'' \frac{1}{\omega''+i\eta-\varepsilon_a} \frac{1}{\omega''-\omega'-i\eta-\varepsilon_i} \frac{1}{\omega-\omega'+\omega''-i\eta-\varepsilon_j} \frac{1}{\omega+\omega''-i\eta-\varepsilon_k}
  $$

  We close the contour in the upper part of the complex plane anti-clockwise. There is a single pole, $\omega'' = \varepsilon_a - i\eta$. The contribution from this integral therefore is

  $$
  -2\pi i \frac{1}{-\omega'-2i\eta+\varepsilon_a-\varepsilon_i} \frac{1}{\omega-\omega'-2i\eta+\varepsilon_a-\varepsilon_j} \frac{1}{\omega-2i\eta+\varepsilon_a-\varepsilon_k}
  $$

For the other terms we obtain accordingly: Second term:

$$\int d\omega'' \frac{1}{\omega''-i\eta-\varepsilon_i}\frac{1}{\omega''-\omega'+i\eta-\varepsilon_a}\frac{1}{\omega-\omega'+\omega''-i\eta-\varepsilon_j}\frac{1}{\omega+\omega''-i\eta-\varepsilon_k}$$

Pole: $\omega''=-i\eta+\varepsilon_a+\omega'$, result:

$$-2\pi i\frac{1}{\omega'-2i\eta+\varepsilon_a-\varepsilon_i}\frac{1}{\omega-2i\eta+\varepsilon_a-\varepsilon_j}\frac{1}{\omega+\omega')-2i\eta+\varepsilon_a-\varepsilon_k}$$

Third term:

$$\int d\omega'' \frac{1}{\omega''-i\eta-\varepsilon_i}\frac{1}{\omega''-\omega'-i\eta-\varepsilon_j}\frac{1}{\omega-\omega'+\omega''+i\eta-\varepsilon_a}\frac{1}{\omega+\omega''-i\eta-\varepsilon_k}$$

Pole: $\omega''=-i\eta+\varepsilon_a-\omega+\omega'$, result:

$$-2\pi i\frac{1}{-\omega+\omega'-2i\eta+\varepsilon_a-\varepsilon_i}\frac{1}{-\omega-2i\eta+\varepsilon_a-\varepsilon_j}\frac{1}{\omega'-2i\eta+\varepsilon_a-\varepsilon_k}$$

Fourth term:

$$\int d\omega'' \frac{1}{\omega''-i\eta-\varepsilon_i}\frac{1}{\omega''-\omega'-i\eta-\varepsilon_j}\frac{1}{\omega-\omega'+\omega''-i\eta-\varepsilon_k}\frac{1}{\omega+\omega''+i\eta-\varepsilon_a}$$

Pole: $\omega''=-i\eta+\varepsilon_a-\omega$, result:

$$-2\pi i\frac{1}{-\omega-i2\eta+\varepsilon_a-\varepsilon_i}\frac{1}{-\omega-\omega'-i2\eta+\varepsilon_a-\varepsilon_j}\frac{1}{-\omega'-i2\eta+\varepsilon_a-\varepsilon_k}$$

After some rearrangements we obtain the contribution from this integral as

$$\begin{aligned}
I^{vooo}=&-2\pi i\frac{1}{(-\omega-i2\eta+\varepsilon_a-\varepsilon_i)(-\omega'-i2\eta+\varepsilon_a-\varepsilon_j)(-\omega-\omega'-i2\eta+\varepsilon_a-\varepsilon_k)}\\
&-2\pi i\frac{1}{(-\omega-2i\eta+\varepsilon_a-\varepsilon_i)(\omega'-2i\eta+\varepsilon_a-\varepsilon_j)(-\omega+\omega'-2i\eta+\varepsilon_a-\varepsilon_k)}\\
&-2\pi i\frac{1}{(\omega-2i\eta+\varepsilon_a-\varepsilon_i)(\omega'-2i\eta+\varepsilon_a-\varepsilon_j)(\omega+\omega'-2i\eta+\varepsilon_a-\varepsilon_k)}\\
&-2\pi i\frac{1}{(\omega-2i\eta+\varepsilon_a-\varepsilon_i)(-\omega'-2i\eta+\varepsilon_a-\varepsilon_j)(\omega-\omega'-2i\eta+\varepsilon_a-\varepsilon_k)}\,.
\end{aligned}$$

The four different terms only differ in the signs of the variables ω and ω'.

- For the four possible contributions with three virtual and a single occupied state we obtain similar terms. Only the sign changes since we have to use a clockwise oriented contour now

and we have to exchange virtual and occupied states:

$$
\begin{aligned}
I^{1o3v} =&2\pi i\frac{1}{(-\omega-i2\eta+\varepsilon_i-\varepsilon_a)(-\omega'-i2\eta+\varepsilon_i-\varepsilon_b)(-\omega-\omega'-i2\eta+\varepsilon_i-\varepsilon_c)}\\
&+2\pi i\frac{1}{(-\omega-2i\eta+\varepsilon_i-\varepsilon_a)(\omega'-2i\eta+\varepsilon_i-\varepsilon_b)(-\omega+\omega'-2i\eta+\varepsilon_i-\varepsilon_c)}\\
&+2\pi i\frac{1}{(\omega-2i\eta+\varepsilon_i-\varepsilon_a)(\omega'-2i\eta+\varepsilon_i-\varepsilon_b)(\omega+\omega'-2i\eta+\varepsilon_i-\varepsilon_c)}\\
&+2\pi i\frac{1}{(\omega-2i\eta+\varepsilon_i-\varepsilon_a)(-\omega'-2i\eta+\varepsilon_i-\varepsilon_b)(\omega-\omega'-2i\eta+\varepsilon_i-\varepsilon_c)}\,.
\end{aligned}
$$

- Now we look at the term

$$
\int d\omega''\frac{1}{\omega''+i\eta-\varepsilon_a}\frac{1}{\omega''-\omega'+i\eta-\varepsilon_b}\frac{1}{\omega-\omega'+\omega''-i\eta-\varepsilon_i}\frac{1}{\omega+\omega''-i\eta-\varepsilon_j}
$$

which has two virtual and two occupied states. we again choose an anti-clockwise contour in the upper half of the complex plane. The integral has two poles here: $\omega''=-i\eta+\varepsilon_a$ and $\omega''=\omega'-i\eta+\varepsilon_b$. All other terms can be obtained analogously. The contributions from all six terms are

$$
\begin{aligned}
I^{2o2v} =&\frac{2\pi i(\varepsilon_a+\varepsilon_b-\varepsilon_i-\varepsilon_j-4i\eta+2\omega)}{(\varepsilon_a-\varepsilon_j-2i\eta+\omega)(\varepsilon_b-\varepsilon_i-2i\eta+\omega)(\varepsilon_a-\varepsilon_i-2i\eta+\omega-\omega')(\varepsilon_b-\varepsilon_j-2i\eta+\omega+\omega')}\\
&+\frac{2\pi i(\varepsilon_a+\varepsilon_b-\varepsilon_i-\varepsilon_j-4i\eta)}{(\varepsilon_a-\varepsilon_j-2i\eta+\omega)(\varepsilon_b-\varepsilon_i-2i\eta-\omega)(\varepsilon_b-\varepsilon_j-2i\eta+\omega')(\varepsilon_a-\varepsilon_i-2i\eta-\omega')}\\
&+\frac{2\pi i(\varepsilon_a+\varepsilon_b-\varepsilon_j-\varepsilon_i-4i\eta-2\omega')}{(\varepsilon_a-\varepsilon_k-2i\eta-\omega')(\varepsilon_b-\varepsilon_j-2i\eta-\omega')(\varepsilon_a-\varepsilon_j-2i\eta+\omega-\omega')(\varepsilon_b-\varepsilon_k-2i\eta-\omega-\omega')}\\
&+\frac{2i\pi(\varepsilon_a+\varepsilon_b-\varepsilon_i-\varepsilon_j-4i\eta+2\omega')}{(\varepsilon_a-\varepsilon_i-2i\eta+\omega')(\varepsilon_b-\varepsilon_j-2i\eta+\omega')(\varepsilon_a-\varepsilon_j-2i\eta+\omega+\omega')(\varepsilon_b-\varepsilon_i-2i\eta-\omega+\omega')}\\
&+\frac{2\pi i(\varepsilon_a+\varepsilon_b-\varepsilon_i-\varepsilon_j-4i\eta)}{(\varepsilon_a-\varepsilon_i-2i\eta+\omega')(\varepsilon_a-\varepsilon_j-2i\eta+\omega)(\varepsilon_b-\varepsilon_i-2i\eta-\omega)(\varepsilon_b-\varepsilon_j-2i\eta-\omega')}\\
&+\frac{2\pi i(\varepsilon_a+\varepsilon_b-\varepsilon_i-\varepsilon_j-4i\eta-2\omega)}{(\varepsilon_a-\varepsilon_j-2i\eta-\omega)(\varepsilon_b-\varepsilon_i-2i\eta-\omega)(\varepsilon_a-\varepsilon_i-2i\eta-\omega+\omega')(\varepsilon_b-\varepsilon_j-2i\eta-\omega-\omega')}
\end{aligned}
$$

d. Beyond-RPA with static interaction For a static interaction, we can also integrate out ω'. In this case $P^{(0)^{(1)}}$ becomes

$$
\begin{aligned}
P^{(0)^{(1)}}_{ia,jb}(\omega) = -\Bigg\{&\frac{(ib|W_0|ja)}{(\varepsilon_a-\varepsilon_i-2i\eta+\omega)(\varepsilon_b-\varepsilon_j-2i\eta-\omega)}\\
&+\frac{(ib|W_0|ja)}{(\varepsilon_a-\varepsilon_i-2i\eta-\omega)(\varepsilon_b-\varepsilon_j-2i\eta+\omega)}\\
&+\frac{(ij|W_0|ab)}{(\varepsilon_a-\varepsilon_i-2i\eta+\omega)(\varepsilon_b-\varepsilon_j-2i\eta+\omega)}\\
&+\frac{(ij|W_0|ab)}{(\varepsilon_a-\varepsilon_i-2i\eta-\omega)(\varepsilon_b-\varepsilon_j-2i\eta-\omega)}\Bigg\}
\end{aligned}
\tag{48}
$$

It fulfills $P^{(0)^{(1)}}(i\omega) = P^{(0)^{(1)}}(-i\omega)$ and comparison to (35) shows that this expression indeed describes the first-order interaction of electron-hole pairs via a Coulomb interaction. Comparison with the solution of the static BSE shows that the contribution containing the integrals $(ij|W_0|ab)$ correspond to resonant transitions (A matrix) and the integrals containing $(ij|W_0|ba)$ to anti-resonant ones (B matrix): In standard notation

$$A_{ia,jb} = -\,\delta_{ij}\delta_{ab}(\varepsilon_a - \varepsilon_i) + (ia|v|jb) - (ij|v|ab)$$
$$B_{ia,jb} = -\,\delta_{ij}\delta_{ab}(\varepsilon_a - \varepsilon_i) + (ia|v|jb) - (ib|v|ja)\ .$$

The imaginary frequency counterpart of (48) is

$$P^{(0)^{(1)}}_{ia,jb}(i\omega) = -\left\{ \frac{(ib|v|ja)}{(\varepsilon_a - \varepsilon_i - i\omega)(\varepsilon_b - \varepsilon_j + i\omega)} + \frac{(ib|v|ja)}{(\varepsilon_a - \varepsilon_i + i\omega)(\varepsilon_b - \varepsilon_j - i\omega)} \right. \\ \left. + \frac{(ij|v|ab)}{(\varepsilon_a - \varepsilon_i + i\omega)(\varepsilon_b - \varepsilon_j + i\omega)} + \frac{(ij|v|ab)}{(\varepsilon_a - \varepsilon_i - i\omega)(\varepsilon_b - \varepsilon_j - i\omega)} \right\}\ . \tag{49}$$

Also this expression is symmetric in $i\omega$ and real. This is best seen if one rewrites it as

$$P^{(0)^{(1)}}_{ia,jb}(i\omega) = -\,2(ib|v|ja)\frac{(\varepsilon_a - \varepsilon_i)(\varepsilon_b - \varepsilon_j) + \omega^2}{[(\varepsilon_a - \varepsilon_i)^2 + \omega^2]\left[(\varepsilon_b - \varepsilon_j)^2 + \omega^2\right]} \\ -\,2(ij|v|ab)\frac{(\varepsilon_a - \varepsilon_i)(\varepsilon_b - \varepsilon_j) - \omega^2}{[(\varepsilon_a - \varepsilon_i)^2 + \omega^2]\left[(\varepsilon_b - \varepsilon_j)^2 + \omega^2\right]}\ . \tag{50}$$

This expression contains double poles related to the loss of the positive definiteness of the corresponding spectral function discussed in ref. 12. Here we are however only interested in the modifications of the screening due to the first-order vertex correction.

After we have calculated $P^{(0)^{(1)}}(i\omega)_{ia,jb}$ we transform it to an auxiliary basis,

$$P^{(0)^{(1)}}(i\omega)_{\alpha\beta} = \sum_{ia}\sum_{jb} c_{ia,\alpha} P^{(0)^{(1)}}(i\omega)_{ia,jb} c_{jb,\beta}\ . \tag{51}$$

The coefficients c approximately map products of KS orbitals to the auxiliary functions f_α,

$$\phi_p(\boldsymbol{r})\phi_q(\boldsymbol{r}) \approx \sum_\alpha c_{pq,\alpha} f_\alpha(\boldsymbol{r})\ ,$$

a procedure known as density fitting.[13–16] In this basis the RPA contribution is given by the expression

$$P^{(0)^{RPA}}_{\alpha,\beta}(i\omega) = -\sum_a\sum_i c_{ia\alpha}\frac{1}{\varepsilon_a - \varepsilon_i - i\omega} c_{ia\beta} + c.c.\ . \tag{52}$$

We then add up both contributions

$$P^{(0)}_{\alpha,\beta}(i\omega) = P^{(0)^{RPA}}_{\alpha,\beta}(i\omega) + P^{(0)^{(1)}}(i\omega)_{\alpha\beta} \tag{53}$$

and proceed by calculating W according to

$$W_{\alpha\beta}(i\omega) = v_{c_{\alpha\beta}} + \sum_{\gamma\delta} v_{c_{\alpha\gamma}} P^{(0)}_{\gamma\delta}(i\omega) W_{\delta\beta}(i\omega) \ . \tag{54}$$

This W is then used in (34).

S4. TECHNICAL DETAILS

All calculations have been performed with MOLGW[17] and the BAND code[9,18]. All (vertex-corrected) *GW* calculations are performed using the analytical frequency integration expression for the self-energy[17,19,20] so that errors due to numerical integration are avoided.

The calculations for the GW100 set haven been performed using the def2-qzvpp basis set. All results given here using the infinite-order vertex approximations have been obtained with MOLGW. Through comparisons with BAND, we have verified that both codes give the same results within a error margin of a few meV. All calculations using the first-order approximations to the vertex have been performed using the BAND code. Since BAND does not support effective core potentials which are necessary to be consistent with the definition of the def2-qzvpp basis, no results for all systems with 5th row elements have been produced.

The calculations for the linear scenes, coronone, circumcoronone, and silicon clusters have been performed with the BAND code using correlation-consistent Dunning basis sets[21,22] ranging from cc-pVDZ to cc-PVQZ (see section S6 for details). Numerical values for first ionization potentials (IP), electron affinities (EA), and HOMO-LUMO gaps are given in tables S1, S2 and S3.

In all BAND calculations, the 4-center integrals are calculated using the pair-atomic density fitting scheme in the implementation of Ref. 23. The size of the auxiliary basis in this approach can be tuned by a single threshold which we set to $\varepsilon_{aux} = 1 \times 10^{-10}$ in all calculations.

The reference values for gw100 have been taken from Ref. 24 with a few exceptions. Since no value for Xe was provided, we have calculated this value using pySCF.[25] Furthermore, the ΔCCSD(T) values given in Ref. 24 for sulfur dioxide (SO_2) and magnesium oxide (MgO) are incorrect. Therefore, we used the EOM-IP-CCSDT IPs provided in Ref. 24 for those two systems.

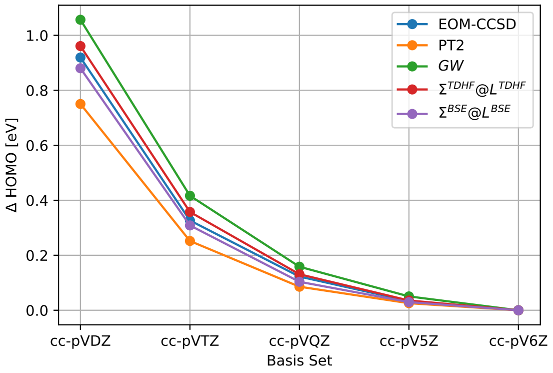

FIG. S1. Convergence of the HOMO energy of water with different methods, calculated using Dunning basis sets. cc-pV6Z serves as reference. All values are in eV.

S5. FURTHER RESULTS FOR GW100

S5.1. Basis set convergence for different methods

S5.2. Vertex corrections for the HOMO of the molecules in GW100

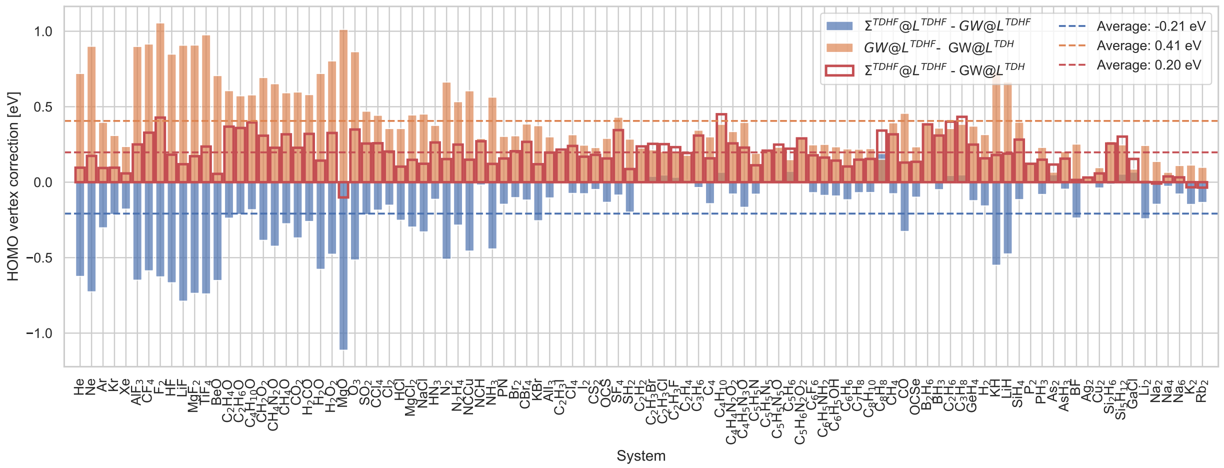

FIG. S2. TDHF Vertex corrections in eV of the highest occupied molecular orbital of the molecules in the GW100 set. Besides the rare gases, the molecules are sorted by decreasing electronegativity of the element most represented in the HOMO.

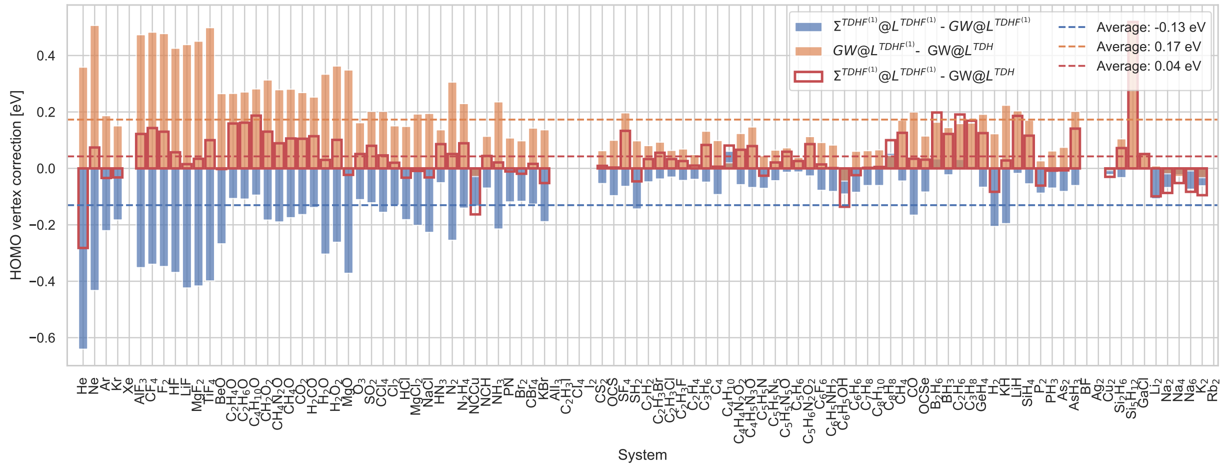

FIG. S3. TDHF$^{(1)}$ Vertex corrections in eV of the highest occupied molecular orbital of the molecules in the GW100 set. Besides the rare gases, the molecules are sorted by decreasing electronegativity of the element most represented in the HOMO.

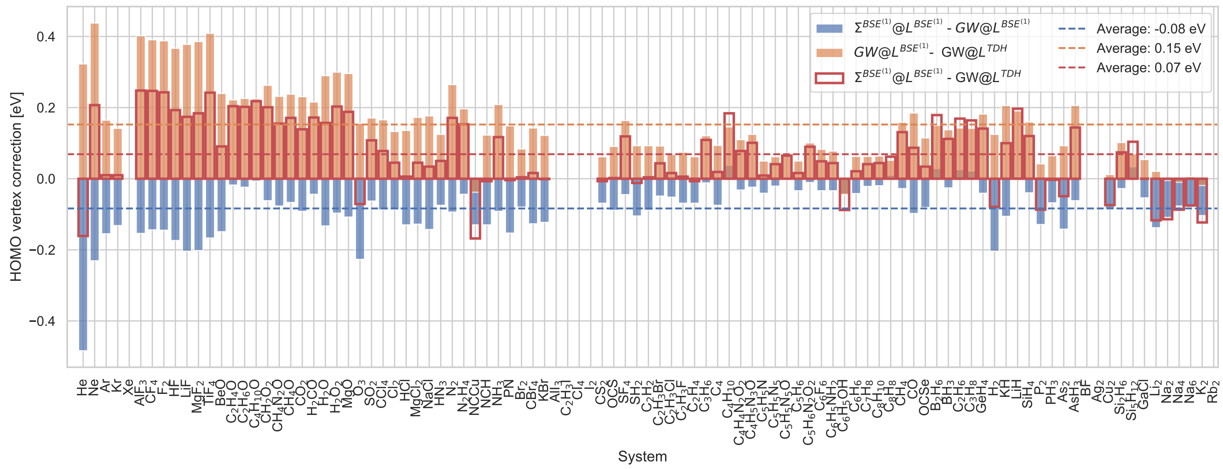

FIG. S4. BSE$^{(1)}$ Vertex corrections in eV of the highest occupied molecular orbital of the molecules in the GW100 set. Besides the rare gases, the molecules are sorted by decreasing electronegativity of the element most represented in the HOMO.

S6. RESULTS FOR LARGE SYSTEMS

Here we give all primary IPs and EAs for linear scenes, coronene, circumcoronene and silicon clusters. All calculations for the linear scenes have been performed using the cc-pvTZ and cc-pVQZ basis sets and the results have been extrapolated to the complete basis set limit using

the expression of Helgaker and coworkers.[26] For Hexacene we only performed the cc-PVTZ calculation and used the differences between cc-pVTZ and cc-pVQZ for tetracene and pentacene to estimate the basis set limit. For coronene and circumcoronene we report here cc-pVDZ results since larger basis sets are prohibitively expense for the latter. The silicon clusters results have been obtained with extrapolation from cc-pVDZ and cc-pTZ.

Molecule	$GW@L^{BSE}$			$\Sigma^{BSE}@L^{BSE}$			$\Sigma^{BSE^{(1)}}@L^{BSE}$		
	IP	EA	gap	IP	EA	gap	IP	EA	gap
C_6H_6	9.58	−1.05	10.63	9.61	−1.48	11.08	9.62	−1.48	11.10
$C_{10}H_8$	8.30	0.20	8.10	8.31	−0.27	8.58	8.28	−0.26	8.55
$C_{14}H_{10}$	7.53	1.03	6.49	7.51	0.55	6.96	7.48	0.56	6.92
$C_{18}H_{12}$	7.01	1.58	5.43	6.98	1.09	5.89	6.95	1.10	5.85
$C_{22}H_{14}$	6.64	1.96	4.68	6.60	1.47	5.14	6.58	1.52	5.06
$C_{26}H_{16}$	6.38	2.24	4.13	6.33	1.75	4.59	6.31	1.80	4.51
Coronene	6.86	−0.04	6.90	6.97	−0.34	7.31	6.96	−0.3	7.29
Circumcoronene	6.01	1.02	4.99	6.09	0.74	5.35	6.07	0.75	5.31
SiH_4	12.66	−3.94	16.59	12.78	−4.01	16.80	12.82	−4.01	16.83
Si_5H_{12}	9.63	−1.50	11.12	9.75	−1.63	11.39	9.74	−1.63	11.38
$Si_{12}H_{26}$	8.57	−0.65	9.23	8.68	−0.80	9.47	8.67	−0.79	9.46
$Si_{26}H_{30}$	8.25	−0.09	8.34	8.34	−0.23	8.57	8.32	−0.23	8.55
$Si_{37}H_{40}$	7.85	0.41	7.44	7.93	0.26	7.67	7.90	0.27	7.63

TABLE S1. $GW@L_{BSE}$, $\Sigma^{BSE^{(1)}}@L_{BSE}$ and $\Sigma^{BSE}@L_{BSE}$ IPs, EAs, and HOMO-LUMO gaps for larger systems. All values are in eV.

Molecule	$GW@L^{TDHF}$			$\Sigma^{TDHF}@L^{TDHF}$			$\Sigma^{TDHF^{(1)}}@L^{TDHF}$		
	IP	EA	gap	IP	EA	gap	IP	EA	gap
C_6H_6	9.52	−0.94	10.46	9.61	−1.42	11.04	9.61	−1.41	11.02
$C_{10}H_8$	8.29	0.30	7.99	8.34	−0.25	8.58	8.20	−0.15	8.35
$C_{14}H_{10}$	7.52	1.11	6.42	7.55	0.53	7.02	7.37	0.72	6.65
$C_{18}H_{12}$	7.01	1.64	5.37	7.03	1.04	5.99	6.80	1.30	5.50
$C_{22}H_{14}$	6.65	2.00	4.65	6.67	1.37	5.30	6.42	1.71	4.71
$C_{26}H_{16}$	6.39	2.27	4.12	6.42	1.61	4.81	6.14	2.01	4.12
Coronene	6.82	0.05	6.78	6.97	−0.32	7.30	6.92	−0.28	7.20
Circumcoronene	5.97	1.10	4.88	6.08	0.74	5.34	6.03	0.81	5.22
SiH_4	12.57	−3.89	16.46	12.71	−3.97	16.68	12.79	−3.97	16.76
Si_5H_{12}	9.54	−1.41	10.95	9.71	−1.55	11.26	9.70	−1.54	11.24
$Si_{12}H_{26}$	8.47	−0.54	9.01	8.62	−0.70	9.32	8.56	−0.70	9.26
$Si_{26}H_{30}$	8.13	0.04	8.08	8.28	−0.13	8.40	8.22	−0.12	8.34
$Si_{37}H_{40}$	7.73	0.55	7.18	7.87	0.37	7.51	7.80	0.39	7.42

TABLE S2. $GW@L_{TDHF}$, $\Sigma^{TDHF^{(1)}}@L_{TDHF}$ and $\Sigma^{TDHF}@L_{TDHF}$ IPs, EAs, and HOMO-LUMO gaps for larger systems. All values are in eV.

Molecule	$GW@L^{TDH}$		
	IP	EA	gap
C_6H_6	9.74	−1.30	11.04
$C_{10}H_8$	8.41	−0.03	8.44
$C_{14}H_{10}$	7.61	0.83	6.78
$C_{18}H_{12}$	7.08	1.40	5.68
$C_{22}H_{14}$	6.71	1.79	4.92
$C_{26}H_{16}$	6.44	2.08	4.36
Coronene	6.97	−0.21	7.18
Circumcoronene	6.09	0.88	5.21
SiH_4	12.96	−4.09	17.06
Si_5H_{12}	9.86	−1.73	11.59
$Si_{12}H_{26}$	8.78	−0.90	9.68
$Si_{26}H_{30}$	8.45	−0.32	8.77
$Si_{37}H_{40}$	8.03	0.19	7.84

TABLE S3. GW@RPA IPs, EAs, and HOMO-LUMO gaps for larger systems. All values are in eV.

REFERENCES

[1] L. Hedin, "New method for calculating the one-particle Green's function with application to the electron-gas problem," Phys. Rev. **139**, A796 (1965).

[2] P. Romaniello, D. Sangalli, J. A. Berger, F. Sottile, L. G. Molinari, L. Reining, and G. Onida, "Double excitations in finite systems," J. Chem. Phys. **130**, 044108 (2009).

[3] G. Strinati, "Application of the Green's functions method to the study of the optical properties of semiconductors," La Riv. Del Nuovo Cim. Ser. 3 **11**, 1–86 (1988).

[4] R. M. Martin, L. Reining, and D. M. Ceperley, *Interacting electrons* (Cambridge University Press, 2016).

[5] E. Maggio and G. Kresse, "GW Vertex Corrected Calculations for Molecular Systems," J. Chem. Theory Comput. **13**, 4765–4778 (2017), arXiv:1706.01815.

[6] P. Romaniello, F. Bechstedt, and L. Reining, "Beyond the GW approximation: Combining correlation channels," Phys. Rev. B **85**, 155131 (2012), arXiv:1204.4463.

[7] R. Orlando, P. Romaniello, and P. F. Loos, "The three channels of many-body perturbation theory: GW, particle-particle, and electron-hole T-matrix self-energies," J. Chem. Phys. **159**, 184113 (2023), arXiv:2309.04167.

[8] M. Rohlfing and S. G. Louie, "Electron-hole excitations and optical spectra from first principles," Phys. Rev. B **62**, 4927–4944 (2000).

[9] F. Bruneval and A. Förster, "Fully dynamic G3W2 self-energy for finite systems: Formulas and benchmark," J. Chem. Theory Comput. **20**, 3218-3230 (2024), arXiv:2401.12892.

[10] X. Ren, N. Marom, F. Caruso, M. Scheffler, and P. Rinke, "Beyond the GW approximation: A second-order screened exchange correction," Phys. Rev. B - Condens. Matter Mater. Phys. **92**, 081104(R) (2015).

[11] R. Kuwahara, Y. Noguchi, and K. Ohno, "GW Γ + Bethe-Salpeter equation approach for photoabsorption spectra: Importance of self-consistent GW Γ calculations in small atomic systems," Phys. Rev. B **94**, 121116(R) (2016).

[12] A. M. Uimonen, G. Stefanucci, Y. Pavlyukh, and R. van Leeuwen, "Diagrammatic expansion for positive density-response spectra: Application to the electron gas," Phys. Rev. B - Condens. Matter Mater. Phys. **91**, 115104 (2015).

[13] E. J. Baerends, D. E. Ellis, and P. Ros, "Self-consistent molecular Hartree—Fock—Slater calculations I. The computational procedure," Chem. Phys. **2**, 41–51 (1973).

[14]B. I. Dunlap, J. W. Connolly, and J. R. Sabin, "On some approximations in applications of Xα theory," J. Chem. Phys. **71**, 3396–3402 (1979).

[15]B. I. Dunlap, J. W. Connolly, and J. R. Sabin, "On first-row diatomic molecules and local density models," J. Chem. Phys. **71**, 4993–4999 (1979).

[16]M. Feyereisen, G. Fitzgerald, and A. Komornicki, "Use of approximate integrals in ab initio theory. An application in MP2 energy calculations," Chem. Phys. Lett. **208**, 359–363 (1993).

[17]F. Bruneval, T. Rangel, S. M. Hamed, M. Shao, C. Yang, and J. B. Neaton, "MOLGW 1: Many-body perturbation theory software for atoms, molecules, and clusters," Comput. Phys. Commun. **208**, 149–161 (2016).

[18]G. Te Velde and E. J. Baerends, "Precise density-functional method for periodic structures," Phys. Rev. B **44**, 7888–7903 (1991).

[19]M. L. Tiago and J. R. Chelikowsky, "Optical excitations in organic molecules, clusters, and defects studied by first-principles Green's function methods," Phys. Rev. B **73**, 205334 (2006), arXiv:0605248 [cond-mat].

[20]M. J. Van Setten, F. Weigend, and F. Evers, "The GW-method for quantum chemistry applications: Theory and implementation," J. Chem. Theory Comput. **9**, 232–246 (2013).

[21]T. H. Dunning, "Gaussian basis sets for use in correlated molecular calculations. I. The atoms boron through neon and hydrogen," J. Chem. Phys. **90**, 1007–1023 (1989).

[22]T. H. Dunning, "A road map for the calculation of molecular binding energies," J. Phys. Chem. A **104**, 9062–9080 (2000).

[23]E. Spadetto, P. H. T. Philipsen, A. Förster, and L. Visscher, "Toward Pair Atomic Density Fitting for Correlation Energies with Benchmark Accuracy," J. Chem. Theory Comput. **19**, 1499–1516 (2023), arXiv:2211.16310.

[24]L. Monzel, C. Holzer, and W. Klopper, "Natural virtual orbitals for the GW method in the random-phase approximation and beyond," J. Chem. Phys. **158**, 144102 (2023).

[25]Q. Sun, X. Zhang, S. Banerjee, P. Bao, M. Barbry, N. S. Blunt, N. A. Bogdanov, G. H. Booth, J. Chen, Z. H. Cui, J. J. Eriksen, Y. Gao, S. Guo, J. Hermann, M. R. Hermes, K. Koh, P. Koval, S. Lehtola, Z. Li, J. Liu, N. Mardirossian, J. D. McClain, M. Motta, B. Mussard, H. Q. Pham, A. Pulkin, W. Purwanto, P. J. Robinson, E. Ronca, E. R. Sayfutyarova, M. Scheurer, H. F. Schurkus, J. E. Smith, C. Sun, S. N. Sun, S. Upadhyay, L. K. Wagner, X. Wang, A. White, J. D. Whitfield, M. J. Williamson, S. Wouters, J. Yang, J. M. Yu, T. Zhu, T. C. Berkelbach, S. Sharma, A. Y. Sokolov, and G. K. L. Chan, "Recent developments in the PySCF program package," J.

Chem. Phys. **153**, 024109 (2020), arXiv:2002.12531.

[26] A. Halkier, T. Helgaker, P. Jørgensen, W. Klopper, H. Koch, J. Olsen, and A. K. Wilson, "Basis-set convergence in correlated calculations on Ne, N2 , and H2O," Chem. Phys. Lett. **286**, 243–252 (1998).
