# Supplementary material for: Why Does the GW Approximation Give Accurate Quasiparticle Energies? The Cancellation of Vertex Corrections Quantified
Source: J Phys Chem Lett. 2024 Dec 13;15(51):12526–34. doi: 10.1021/acs.jpclett.4c03126 (PMC11684030; doi:10.1021/acs.jpclett.4c03126)
Supplement: Supplementary file 3 — jz4c03126_si_003.pdf [file jz4c03126_si_003.pdf]

jz-2024-03126z.R1

Name: Peer Review Information for "Why does the GW approximation give accurate quasiparticle energies? The cancellation of vertex corrections quantified"

First Round of Reviewer Comments

Reviewer: 1

Comments to the Author

In this manuscript the authors study the cancellation between internal (correction to W) and external (correction to the self-energy) vertex corrections by exploring various non-local vertex approximations beyond the GWA for molecular systems of the GW100 set. This cancellation, which has been indicated in literature as at the origin of the GWA success, is now quantified in this work. The manuscript also studies vertex corrections for the HOMO and LUMO energies of molecules of systematically increasing size and it points out the importance of including screened vertex corrections for large systems.

This is a well-done and systematic study, carried out using two well-known codes (MOLGW and BAND) to check the quality and consistency of the results, and with a clever used of the essential equations to implement. I believe that it meets the standard for publication in The Journal of Physical Chemistry Letters.

However, it would be useful if the authors could address my questions/comments, which are reported in the following.

1) The quality of the GW and beyond GW methods are checked by comparing the results for the HOMO energy of the GW100 set with the corresponding value obtained using CCSD(T). The def2-qzvpp basis set is used for all calculations. I know that the use of the same basis set used in the reference method is a common practice when accessing the accuracy of a given method, but according to me it would be better to compare the converged (with respect to the basis set) result. However, the def2-qzvpp is a valence QZ so I guess that all the GW and beyond GW results are converged. Is this the case?

2) The vertex cancellation is checked for the HOMO and LUMO, which occupied a special place in spectroscopy, but of course the spectrum is much richer than that. Do the authors think that

similar trends will be observed also for the other occupied and unoccupied orbitals? And if yes, why?

3) In the abstract the authors write “We finally consider increasingly large clusters

and extrapolate that our conclusions would hold for extended systems”. Which conclusions do they extrapolate to extended systems? The fact that screened vertex corrections are important? This is not clear from the manuscript. Moreover the study on larger clusters is done starting from G^HF, which is different from what it is commonly done in extended systems where one prefers a KS starting point. Do the authors think that this could influence their extrapolation to extended systems?

4) In the main manuscript it is written that all calculations for the GW100 set have been carried out using a def2-qzvpp basis set, but there is nothing reported for the larger systems (except in the SI). It would be useful to have this information in the main manuscript as well.

5) A typo in the SI: after Eq. (4) “For first term”--→”The first term”

6) If I am not mistaken Eq. (14) was previously reported in PRB 2012, 85, 155131 (already cited by the authors in the main manuscript) and, for example, in the book “Interacting electrons. Theory and computational approaches. Cambridge University press, 2016” . It would be fair to cite these references for Eq. (14).

Reviewer: 2

Comments to the Author

The manuscript discusses vertex correction to the widely used GW method for single-electron spectra of molecules, in particular the HOMO and LUMO energies. With help of the irreducible kernel, the authors arrive at expressions for the two-particle correlation function and for the self energy, both of which can then be calculated to infinite order provided that a reasonable approximation for the kernel is employed. This goes far beyond existing GW approaches and constitutes a significant contribution to systematic improvement of single-particle spectra.

The authors investigate several possibilities for the kernel both for the two-particle correlation function and for the self energy and observe, not surprisingly, that the same kernel should be uswed

for both quantities, and that the most advanced kernel yields the best results (in comparison to CCSD(T) calculations used as reference).

The paper, although appearing rather technical, is timely insofar as it carefully addresses a current topic (vertex corrections for molecular systems) and discusses systematically the various contributions, thus providing high-quality reference data. I recommend publication, essentially as is.

I suggest that the authors state in clear terms that Eq. (4) is an approximation, and that its general quality (as high as it may be) is difficult to assess.

Author's Response to Peer Review Comments:

Dear Editor,

We thank you for providing us with these two very positive reviews.
We are happy to address both Reviewers' comments hereafter.

With best regards,

Arno Förster and Fabien Bruneval

Reply to Reviewer 2

Reviewer's comments are in blue.
Changes implemented in the resubmitted manuscript are in red.

Reviewer: 1

Recommendation: This paper is publishable subject to minor revisions noted. Further review is not needed.

Comments:
In this manuscript the authors study the cancellation between internal (correction to W) and external (correction to the self-energy) vertex corrections by exploring various non-local vertex approximations beyond the GWA for molecular systems of the GW100 set. This cancellation, which has been indicated in literature as at the origin of the GWA success, is now quantified in this work. The manuscript also studies vertex corrections for the HOMO and LUMO energies of molecules of systematically increasing size and it points out the importance of including screened vertex corrections for large systems.
This is a well-done and systematic study, carried out using two well-known codes (MOLGW and BAND) to check the quality and consistency of the results, and with a clever used of the essential equations to implement. I believe that it meets the standard for publication in The Journal of Physical Chemistry Letters.

We thank the reviewer for their positive recommendation.

However, it would be useful if the authors could address my questions/comments, which are reported in the following.

1) The quality of the GW and beyond GW methods are checked by comparing the results for the HOMO energy of the GW100 set with the corresponding value obtained using CCSD(T). The def2-qzvpp basis set is used for all calculations. I know that the use of the same basis set used in the reference method is a common practice when accessing the accuracy of a given method, but according to me it would be better to compare the converged (with respect to the basis set) result. However, the def2-qzvpp is a valence QZ so I guess that all the GW and beyond GW results are converged. Is this the case?

We agree with the reviewer here, it would always be best to perform benchmarks at the complete basis set limit. However, as the reviewer already guessed, def2-qzvpp is so large, that essentially no significant basis set error remains. The remaining basis set errors between (vertex-corrected) GW and CCSD(T) will essentially cancel since the quasiparticle energies calculated with both methods will converge with the same sign. We have also verified that vertex-corrected GW calculations do converge to the complete basis set limit significantly faster than GW, at a rate which is equal to CCSD(T). We added a plot to the supporting information, which illustrates this, using the Water molecule as an example. Therefore, at the def2-qzvpp level, our calculations will be mostly free of basis set errors.

2) The vertex cancellation is checked for the HOMO and LUMO, which occupied a special place in spectroscopy, but of course the spectrum is much richer than that. Do the authors think that similar trends will be observed also for the other occupied and unoccupied orbitals? And if yes, why?

This is for sure an important question, but since we did not do such calculations at the moment, we leave this for a future study.

3) In the abstract the authors write “We finally consider increasingly large clusters and extrapolate that our conclusions would hold for extended systems”. Which conclusions do they extrapolate to extended systems? The fact that screened vertex corrections are important? This is not clear from the manuscript. Moreover the study on larger clusters is done starting from G^HF, which is different from what it is commonly done in extended systems where one prefers a KS starting point. Do the authors think that this could influence their extrapolation to extended systems?

The reviewer addresses an important point, we have not been clear about this. We indeed wanted to say, that our conclusions about the compensation of vertex corrections will also hold for extended systems. This is what we observe in our cluster calculations. We also believe that our observation on the importance of screened interactions will carry over to extended systems, but this is. Not what we wanted to emphasize in our abstract. In our revised manuscript, we changed the last sentence of our abstract, to clarify this point.

Then, the reviewer mentions a second point: We indeed exclusively worked with HF Green’s function. We have done this for two reasons. First, we wanted to avoid adjustments of the starting point, and second, at least for smaller molecules, HF orbitals, and eigenvalues are relatively close to fully self-consistent GW eigenvalues and eigenvectors. The second point will not be valid anymore for extended systems, and the reviewer rightly points out, that a KS starting point would potentially be more appropriate. We have not explored the dependence of our results on the KS starting point, but we are planning to revisit this eventually in a follow-up paper. While changing the starting point will of course change our numbers, we do however not believe, that our conclusions about the cancellations of the vertex corrections will be affected.

4) In the main manuscript it is written that all calculations for the GW100 set have been carried out using a def2-qzvpp basis set, but there is nothing reported for the larger systems (except in the SI). It would be useful to have this information in the main manuscript as well.

We agree with the reviewer, that some information should be added also to the main text. We did not mention the basis sets used for these calculations in the main text since they changed from system to system, since for some calculations we could not afford to use large basis sets, and the description of the basis sets would become very lengthy. In our revised manuscript, we added a sentence, stating that we used different basis sets, and refer to the supporting information for details.

5) A typo in the SI: after Eq. (4) “For first term”--→”The first term”

We have corrected this typo in our revised manuscript

6) If I am not mistaken Eq. (14) was previously reported in PRB 2012, 85, 155131 (already cited by the authors in the main manuscript) and, for example, in the book “Interacting electrons. Theory and computational approaches. Cambridge University press, 2016” . It would be fair to cite these references for Eq. (14).

We agree with the reviewers, that we should have cited this reference also in our supporting information. We have added the references from the main text also to our revised supporting information.

Reviewer: 2

Recommendation: This paper is publishable subject to minor revisions noted. Further review is not needed.

Comments:
The manuscript discusses vertex correction to the widely used GW method for single-electron spectra of molecules, in particular the HOMO and LUMO energies. With help of the irreducible kernel, the authors arrive at expressions for the two-particle correlation function and for the self energy, both of which can then be calculated to infinite order provided that a reasonable approximation for the kernel is employed. This goes far beyond existing GW approaches and constitutes a significant contribution to systematic improvement of single-

particle spectra.

The authors investigate several possibilities for the kernel both for the two-particle correlation function and for the self energy and observe, not surprisingly, that the same kernel should be uswed for both quantities, and that the most advanced kernel yields the best results (in comparison to CCSD(T) calculations used as reference).

The paper, although appearing rather technical, is timely insofar as it carefully addresses a current topic (vertex corrections for molecular systems) and discusses systematically the various contributions, thus providing high-quality reference data. I recommend publication, essentially as is.

We thank the reviewer for their very positive review.

I suggest that the authors state in clear terms that Eq. (4) is an approximation, and that its general quality (as high as it may be) is difficult to assess.

We agree with the reviewer, that we should be clear about this. We have clarified this point in our revised manuscript.
